# Association of Carotid Plaque Lp-PLA_2_ with Macrophages and *Chlamydia pneumoniae* Infection among Patients at Risk for Stroke

**DOI:** 10.1371/journal.pone.0011026

**Published:** 2010-06-09

**Authors:** Berna Atik, S. Claiborne Johnston, Deborah Dean

**Affiliations:** 1 Center for Immunobiology and Vaccine Development, Children's Hospital Oakland Research Institute, Oakland, California, United States of America; 2 Department of Neurology, University of California San Francisco, San Francisco, California, United States of America; 3 Department of Medicine, University of California San Francisco, San Francisco, California, United States of America; 4 Department of Bioengineering, University of California, Berkeley, California, United States of America; 5 Department of Bioengineering, University of California San Francisco, San Francisco, California, United States of America; University of California Merced, United States of America

## Abstract

**Background:**

We previously showed that the burden of *Chlamydia pneumoniae* in carotid plaques was significantly associated with plaque interleukin (IL)-6, and serum IL-6 and C-reactive protein (CRP), suggesting that infected plaques contribute to systemic inflammatory markers in patients with stroke risk. Since lipoprotein-associated phospholipase A2 (Lp-PLA_2_) mediates inflammation in atherosclerosis, we hypothesized that serum Lp-PLA_2_ mass and activity levels and plaque Lp-PLA_2_ may be influenced by plaque *C. pneumoniae* infection.

**Methodology/Principal Findings:**

Forty-two patients underwent elective carotid endarterectomy. Tissue obtained at surgery was stained by immunohistochemistry for Lp-PLA_2_ grade, macrophages, IL-6, *C. pneumoniae* and CD4+ and CD8+ cells. Serum Lp-PLA_2_ activity and mass were measured using the colorimetric activity method (CAM™) and ELISA, respectively. Serum homocysteine levels were measured by HPLC. Eleven (26.2%) patients were symptomatic with transient ischemic attacks. There was no correlation between patient risk factors (smoking, coronary artery disease, elevated cholesterol, diabetes, obesity, hypertension and family history of genetic disorders) for atherosclerosis and serum levels or plaque grade for Lp-PLA_2_. Plaque Lp-PLA_2_ correlated with serum homocysteine levels (p = 0.013), plaque macrophages (p<0.01), and plaque *C. pneumoniae* (p<0.001), which predominantly infected macrophages, co-localizing with Lp-PLA_2_.

**Conclusions:**

The significant association of plaque Lp-PLA_2_ with plaque macrophages and *C. pneumoniae* suggests an interactive role in accelerating inflammation in atherosclerosis. A possible mechanism for *C. pneumoniae* in the atherogenic process may involve infection of macrophages that induce Lp-PLA_2_ production leading to upregulation of inflammatory mediators in plaque tissue. Additional *in vitro* and *in vivo* research will be needed to advance our understanding of specific *C. pneumoniae* and Lp-PLA_2_ interactions in atherosclerosis.

## Introduction

Carotid atherosclerosis is a major risk factor for an ischemic stroke [1]. While lipid metabolism and inflammation have been the major focus of atherosclerosis research for many years, there has been a growing interest in lipoprotein-associated phospholipase A2 (Lp-PLA_2_) because it is a key enzyme both in lipid metabolism and in stimulating inflammation [2].

Lp-PLA_2_ is a calcium-independent member of the phospholipase A2 enzyme family. Monocytes, macrophages, T-lymphocytes, mast cells and liver cells are the main sources for Lp-PLA_2_ [3], [4]. It is carried primarily by low-density lipoprotein (LDL). Lp-PLA_2_ catalyzes the hydrolysis of oxidized LDL, which produces proinflammatory mediators lysophosphatidylcholine (LysoPC) and oxidized fatty acid (oxFA) [5].

Many clinical studies have found an association between increasing serum levels of Lp-PLA_2_ mass and/or activity at the time of a cardiovascular incident in addition to an elevated risk of mortality and morbidity over time [6], [7], [8]. One study showed that Lp-PLA_2_ mRNA and protein levels were six times higher in atherosclerotic lesions compared to normal tissue samples [9].

Cumulative evidence suggests that *C. pneumoniae* also plays an important role in atherosclerosis [10], [11], [12], [13], [14]. The organism is thought to infect pulmonary monocytes that are then transported via the vasculature to localize in arteries where infection can spread [15]. *C. pneumoniae* is a ubiquitous pathogen that frequently causes upper and lower respiratory tract infections worldwide [16]. More than half of the patients with atherosclerosis have evidence for *C. pneumoniae* infection based on a variety of studies using detection methods such as immunohistochemistry (IHC) and electron microscopy of plaques [17], [18], PCR or real time RT-PCR of DNA/RNA extracted from plaques [18], [19], [21], and seroepidemiologic analyses among different populations [20], [21]. Other studies have shown viable organisms in the carotid arteries of stroke patients [19], [22] and patients with CAD [11], [23]. Furthermore, recent studies in murine and rabbit models suggest that *C. pneumoniae* can target the vasculature, induce inflammation and initiate or promote the development of atherosclerosis [14], [24], [25]. In the same models, *C. pneumoniae* accelerated atherosclerotic development, while treatment with azithromycin prevented the disease [12], [14]. However, treatment did not have the same effect on chronically infected mice [26], where organism persistence may have contributed to resistance to therapy. Recent *in vitro* studies also strongly suggest a role for *C. pneumoniae* in the genesis and progression of atherosclerosis [27], [28]

More recently, we have shown that the burden of *C. pneumoniae* infection was significantly associated with up-regulation of plaque interleukin (IL)-6 expression, which correlated with elevated serum levels of IL-6 and C-reactive protein (CRP) [18]. IL-6 stimulates liver CRP production, an acute phase reactant associated with risk of myocardial infarction (MI) and stroke. IL-6 secretion in *C. pneumoniae*-infected plaques could explain elevated systemic markers of inflammation among individuals at risk for vascular events.

There is currently no research, to our knowledge, correlating serum Lp-PLA_2_ mass and activity levels with plaque Lp-PLA_2_ or the interaction of *C. pneumoniae* infection and Lp-PLA_2_ on arterial disease and inflammation. We hypothesized that serum Lp-PLA_2_ mass and activity levels as well as plaque Lp-PLA_2_ would be significantly elevated in the presence of plaque *C. pneumoniae* infection, suggesting an interactive role in accelerating inflammation in atherosclerosis.

## Materials and Methods

### Ethics Statement

The University of California at San Francisco (UCSF) and Children's Hospital Oakland Research Institute (CHRCO) Institutional Review Board committees approved the study. Informed written consent was obtained for all study subjects. The study was conducted according to the principles of the Declaration of Helsinki.

### Study subjects

In this cross-sectional study, subjects underwent elective carotid endarterectomy at UCSF, as described previously [19]. The treated carotid artery was associated with signs and/or symptoms of neurologic disease.

### Lipoprotein-associated phospholipase A2 (Lp-PLA_2_) detection by immunohistochemistry (IHC) in carotid artery tissue

Lp-PLA_2_ was detected by IHC using three, five-micron sections per carotid plaque in optimal cutting temperature (OCT) medium. The carotid plaque tissue was stored at −80°C in OCT until sectioning. Briefly, each section was blocked with casein (Biocare Medical, Concord, CA) and stained with anti-Lp-PLA_2_ monoclonal antibody (diaDexus, Inc., South San Francisco, CA) diluted 1∶400 in diluent (Biocare). Samples were washed with TBS, blocked with avidin (Biocare), washed again and blocked with biotin (Biocare) prior to applying goat, anti-mouse IgG antibody (Biocare). Streptavidin was applied followed by alkaline phosphatase chromagen-fast red (Biocare). The section was counterstained with hematoxylin to detect each cell. In independent experiments, excess primary antibody and, separately, excess secondary antibody was used on adjacent sections to ensure no non-specific staining of either antibody for Lp-PLA_2_. In addition, a mouse non-immune IgG (Biocare) was used as a final negative control.

**Figure 1:**
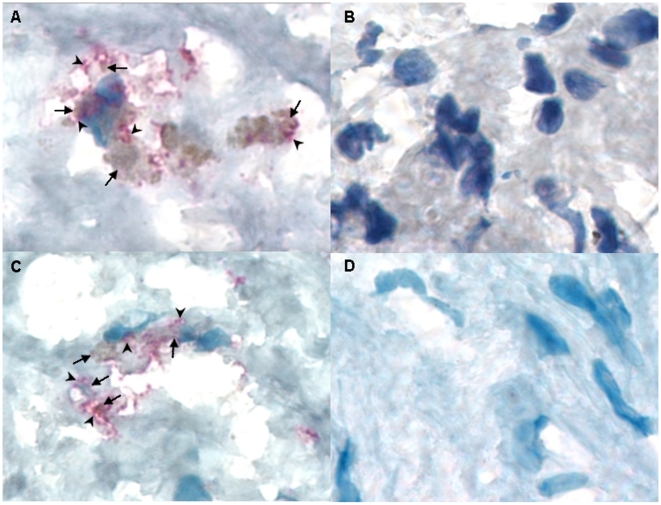
Carotid plaque sections showing co-localization of Lp-PLA_2_ and *C. pneumoniae*, and macrophages and *C. pneumoniae*. A) Lipoprotein-associated phospholipase A2 (Lp-PLA_2_) was detected by Immunohistochemistry (IHC) using anti- Lp-PLA_2_ specific monoclonal antibody (diaDexus) and chromagen fast red (arrowheads); *C. pneumoniae* was detected by IHC using a CHsp60-specific MAb and chromagen DAB (arrows); ×1000. B) Negative control of a positive carotid plaque section (same patient as in A); ×1000. C) Carotid plaque section showing co-localization of *C. pneumoniae* (DAB; arrows) and macrophages detected by IHC using CD68 macrophage-specific monoclonal antibody and chromagen fast red (arrowheads); ×1000. D) Negative control of a positive carotid plaque section (same patient as in C).

Using light microscopy at 400×, samples were read independently by two individuals who were blinded to all patient data. Samples were graded based on percentage of the tissue staining for Lp-PLA_2_ for the entire plaque section. We used 1, 2 or 3+ for the entire carotid section for each patient sample (3 sections per patient carotid sample) where a grade of ≥1 was considered positive for Lp-PLA_2_; 1, 1–25% of the tissue; 2, 26–50% of the tissue; 3, >50% of the tissue. The three sections from each carotid sample were used to determine the within-sample variation, and the average of the three was used for analysis. Because of the ease of visualization of the staining for Lp-PLA_2_ (see Fig 1A), software was not required for quantitation.

All sections that stained positive for Lp-PLA_2_ were probed with chlamydial-specific heat shock protein 60 (CHsp60) MAb (Affinity Bioreagents) to determine the precise co-localization of Lp-PLA_2_ and infection using the methods as described above except that a horseradish peroxidase–conjugated secondary antibody with chromogen diaminobenzidine (DAB; Biocare) was used to detect chlamydiae. Positive and negative controls were as described previously [18], [19]. All three sections for each patient were read in entirety and analyzed as described for Lp-PLA_2_ except that the cells were counted to determine the number with Lp-PLA_2_ alone, *C. pneumoniae* alone, and the number with both for each section as quantitative measures.

Two additional adjacent sections for each patient sample were used to co-localize *C. pneumoniae* with macrophages. The sections were stained for *C. pneumoniae* as above, and macrophages were stained with fast red as for Lp-PLA_2_ except that the primary monoclonal antibody was macrophage specific (CD68; Biocare).

While other infectious agents may also be involved in atherosclerosis, the evaluation of these pathogens was beyond the scope of this study.

### Prior data on the patient population used for analyses

In our prior studies, plaque tissues were noted to have a high grade of atherosclerosis [18], [19]. Data on the same population from our previous publications were also used for analyses [18], [19]. These included IHC to detect macrophages, CD4+ cells, CD8+ cells, IL-6, and *C. pneumoniae* in the adjacent sections of the same block of carotid tissue used for Lp-PLA_2_ IHC above. In addition, we previously determined plaque IL-6 gene expression by quantitative (q)RT-PCR, plaque *C. pneumoniae* burden by qRT-PCR, serum C-reactive protein (CRP) levels, and IL-6 serum protein levels, the methods of which are described in detail in our references [18], [19].

### Measurement of serum mass and activity levels for lipoprotein-associated phospholipase A2 (Lp-PLA_2_) and homocysteine levels

All serum biochemical analyses were performed on serum from blood or blood plasma obtained at the time of carotid endarterectomy.

Lp-PLA_2_ mass was determined by ELISA (PLAC® Test; diaDexus) in serum according to the manufacturers instructions using two specific monoclonal antibodies in a 96-well format. Quantitation was calibrated to a recombinant Lp-PLA_2_ antigen standard. The lower detection limit was 2 ng/mL; interassay coefficient of variation (CV) was between 6% and 7%. Lp-PLA_2_ activity levels were measured by CAM™ assay (diaDexus) in serum according to the manufacturers instructions. Samples were analyzed in a 96-well microplate with a colorimetric substrate converted on hydrolysis by phospholipase enzyme. Briefly, 25 µL of sample, standard, or control was added per well, followed by addition of assay buffer plus substrate. Change in absorbance was measured at 405 nm. Lp-PLA_2_ activity in nmol/min/mL was calculated from the slope, based on a standard conversion factor from a p-Nitrophenol calibration curve. Activity levels between 13.5–46.1 were considered as quartile-1, 46.2–69 as quartile-2, 69.1–89.2 as quartile-3, and 89.3–143.2 as quartile-4.

Homocysteine levels in serum (the preferred sample type) were measured by fluorometric high-performance liquid chromatography (HPLC; Quest Diagnostics).

### Statistical analysis

Clinical and laboratory characteristics of patients were compared by serum Lp-PLA_2_ mass and activity levels and Lp-PLA_2_ plaque grade. Before comparing continuous variables for Lp-PLA_2_ plaque grades, the normality assumption was checked by Shapiro-Wilk test and the distributional diagnostic plots for these variables: age, Lp-PLA_2_ serum activity, and homocysteine levels. All except homocysteine had a normal distribution. Square root transformation of homocysteine levels was performed to achieve the normality assumption.

Student t-test was used to compare plaque Lp-PLA_2_ positive vs. negative groups for continuous variables with normal distribution. Pearson chi-square test was used to compare plaque Lp-PLA_2_ positive vs. negative groups for binomial variables: history of smoking, coronary artery disease, elevated cholesterol, diabetes, obesity, hypertension and family history of genetic disorders, elevated serum CRP, serum IL-6, *C. pneumoniae* by qRT-PCR and plaque IL-6, CD4+, CD8+, macrophages, and *C. pneumoniae*. Multiple logistic regression was also performed for these comparisons, including all variables associated with Lp-PLA_2_ (at p<0.20) with a stepwise removal of any that did not contribute (at p>0.10). Since Lp-PLA_2_ serum activity results were categorized into four quartiles, Kruskal-Wallis test was used for comparing serum Lp-PLA_2_ quartiles for clinical characteristics. Nonparametric Spearman Rank test was used to calculate correlation coefficients between variables with Bonferroni adjustment. A P value of <0.05 was considered statistically significant. STATA version-9 (College Station, TX) was used for all analyses.

## Results

### Patient characteristics and association with serum Lp-PLA_2_ activity and plaque Lp-PLA_2_

Characteristics of the 42 study patients are shown in Tables 1 and 2 stratified by Lp-PLA_2_ plaque status and Lp-PLA_2_ serum activity, respectively. We considered the 42 patients to be a representative cohort of patients with neurologic signs and/or symptoms consistent with carotid vascular disease in addition to the fact that they were enrolled consecutively after informed consent from the pre-operative evaluation clinic at UCSF as previously described [18], [19]. None of the patients had stroke but all had neurologic symptoms, indicating carotid ischemia: 30 (71.4%) had symptoms on the left side, 11 (26.2%) on the right, and 1 (2.4%) bilaterally. It should be noted that the treated carotid artery was associated with symptoms on the ipsilateral side. There were no significant correlations of risk factors (smoking, coronary artery disease, elevated cholesterol, diabetes, obesity, hypertension and family history of genetic disorders) or clinical characteristics with Lp-PLA_2_ serum levels or tissue Lp-PLA_2_ grade ≥1 (Tables 1 and 2) or with *C. pneumoniae* infection as defined by qRT-PCR or IHC as described previously [18], [19] (Tables 3 and 4).

**Table 1:** Clinical characteristics of study population by plaque Lp-PLA_2_.^a^

Characteristics	Total (n = 42)	Plaque Lp-PLA_2_ negative (n = 23)	Plaque Lp-PLA_2_ positive (n = 19)	*P* Value^b^
Age (mean +/− s.d.)	72 (9.4)	72.4 (+/−10.6)	72.3 (+/−8)	0.987
Sex:				0.327
Male	30 (71.4%)	15 (65.2%)	15 (78.9%)	
Female	12 (28.6%)	8 (34.8%)	4 (21.1%)	
Smoker	33 (78.6%)	17 (73.9%)	16 (84.2%)	0.418
CAD	23 (54.8%)	11 (47.8%)	12 (63.2%)	0.320
Hypertension history	36 (85.7%)	20 (87%)	16 (84.2%)	0.800
High cholesterol	27 (64.3%)	14 (60.8%)	13 (68.4%)	0.611
Diabetes mellitus	9 (21.4%)	4 (17.4%)	5 (26.3%)	0.483
Symptomatic				0.264
Left side	30 (71.4%)	18 (78.3%)	12 (63.2%)	
Right side	11 (26.2%)	4 (17.4%)	7 (63.7%)	
Both	1 (2.4%)	1 (4.3%)	0	

*Abbreviations:* Lp-PLA_2_, Lipoprotein-associated phospholipase A2; CAD, Coronary Artery Disease.

a Values expressed are for plaque Lp-PLA_2_ grade of ≥1 as the results were the same for any grade ≥1.

b *P* values were generated by chi-square test except for age, where t-test was used for comparison.

**Table 2:** Clinical characteristics of study population by serum Lp-PLA_2_ activity.^a^

Characteristics	Lp-PLA_2_ Activity 1^st^ quartile (n = 10)^c^	Lp-PLA_2_ Activity 2^nd^ quartile (n = 10)^c^	Lp-PLA_2_ Activity 3^rd^ quartile (n = 10)^c^	Lp-PLA_2_ Activity 4^th^ quartile (n = 11)^c^	*P* Value^b^
Age (mean +/− s.d.)	67.9 (+/−10.1)	71 (+/−10.1)	75.9 (+/−7.8)	73.7(+/−8.5)	0.519
Sex:					0.403
Male (n = 29^b^)	8 (27.6%)	5 (17.2%)	8 (27.6%)	8 (27.6%)	
Female (n = 12^b^)	2 (17%)	5 (41%)	2 (17%)	3 (25%)	
Smoker	8 (25%)	7 (21.9%)	6 (25%)	11 (28.1%)	0.916
CAD	2 (9.1%)	7 (31.8%)	6 (27.3%)	7 (31.8%)	0.101
Hypertension	7 (20%)	9 (25.7%)	8 (22.9%)	11 (31.4%)	0.243
High cholesterol	4 (15.4%)	8 (30.8%)	5 (19.2%)	9 (34.6%)	0.115
Diabetes mellitus	3 (33.3%)	1 (11.1%)	1 (11.1%)	4 (44.5%)	0.337
Symptomatic:					0.095
Right side	9 (31%)	7 (24.2%)	4 (13.8%)	9 (31%)	
Left side	1 (9.1%)	2 (18.2%)	6 (54.6%)	2 (18.2%)	
Both sides	0	1 (100%)	0	0	

*Abbreviations:* Lp-PLA_2_, Lipoprotein-associated phospholipase A2; Lp-PLA_2_.

Activity, range measured in nmol/min/mL; CAD, Coronary Artery Disease.

a Lp-PLA_2_ Activity range measured in nmol/min/mL.

b *P* values were generated by chi-square test except for age, where t-test was used for comparison.

c Serum Lp-PLA2 activity information was missing for one person.

**Table 3:** Correlations among plaque characteristics and serum levels of inflammatory markers.

	Carotid Plaque Lp-PLA_2_	Serum Lp-PLA_2_ mass (ng/mL)	Serum Lp-PLA_2_ activity
**Carotid Plaque**			
*Cpn* by qRT-PCR	0.21	−0.28	−0.23
*Cpn* by IHC	0.39^a^	−0.24	−0.08
IL-6 expression	0.23	−0.31^d^	−0.22
IL-6 by IHC	0.11	−0.34^d^	−0.32^d^
Macrophages	0.37^b^	0.06	0.11
CD4+	0.17	−0.04	−0.13
CD8+	−0.02	−0.10	−0.13
B-cell	−0.11	−0.22	−0.32
Lp-PLA_2_	1	0.14	0.19
**Serum**			
Lp-PLA_2_ mass (ng/mL)	0.14	1	0.76^a^
Lp-PLA_2_ activity	0.19	0.76^a^	1
CRP	0.18	−0.25	−0.21
IL-6	0.08	−0.27	−0.19
Homocysteine	0.38^c^	−0.006	0.12

*Abbreviations:* Lp-PLA_2_, Lipoprotein-associated phospholipase A2; Cpn, *C. pneumoniae*; qRT-PCR, quantitative real-time reverse transcription PCR; IL-6, interleukin-6; IHC, immunohistochemistry; CRP, C-reactive protein.

a p<0.001.

b p<0.01.

c p<0.013.

d p<0.05.

**Table 4:** Correlations between serum Lp-PLA_2_ activity, and plaque and serum Lp-PLA_2_ mass.

Serum Lp-PLA_2_ activity (range in nmol/min/mL)	Plaque Lp-PLA_2_ a	Serum Lp-PLA_2_ mass (ng/mL)
1^st^ quartile (13–46.1)	0.04 (p = 0.822)	−0.47 (p< = 0.001)
2^nd^ quartile (46.2–69)	−0.14 (p = 0.375)	−0.31 (p = 0.043)
3^rd^ quartile (69.1–89.2)	0.02 (p = 0.987)	0.37 (p = 0.015)
4^th^ quartile (89.3–143.2)	0.13 (p = 0.417)	0.48 (p = 0.001)

*Abbreviations:* Lp-PLA_2_, Lipoprotein-associated phospholipase A2.

a Values expressed are for plaque Lp-PLA_2_ grade of ≥1 as there were no significant correlations with grades >1.

### Correlation among carotid plaque characteristics and serum levels of inflammatory markers

Serum Lp-PLA_2_ mass and activity levels were significantly correlated (r = 0.76, p = 0.001, Table 3). High Lp-PLA_2_ activity was also correlated with Lp-PLA_2_ mass (r_3_ = 0.37 and r_4_ = 0.48, p_3_ = 0.015 and p_4_ = 0.001, respectively, Table 4).

Interestingly, 94.7% (18/19) of patients who had plaque Lp-PLA_2_ also had plaque *C. pneumoniae*. Plaque Lp-PLA_2_ presence (for all quantitative grades above 1) was significantly correlated with *C. pneumoniae* (r = 0.39, p = 0.001) and macrophages (r = 0.37, p = 0.01, Table 3), and with higher serum homocysteine levels (r = 0.38, p = 0.013, Table 3). Plaque Lp-PLA_2_ co-localized with *C. pneumoniae*, macrophages and CD4+ lymphocytes by IHC in the shoulder and necrotic core of the plaques as has been noted by others [9]. We found that 52% of cells showed evidence for Lp-PLA_2_ protein and infection with *C. pneumoniae* (Figure 1A). In addition, 39% of macrophages were infected with *C. pneumoniae* (Figure 1B).

In Table 3, the correlation between carotid plaque Lp-PLA_2_ and plaque IL-6 expression, IL-6 detected by IHC, serum IL-6, and CRP was statistically insignificant for all plaque Lp-PLA_2_ grades. Serum Lp-PLA_2_ mass levels were negatively correlated with plaque IL-6 expression and IL-6 detected by IHC (r = −0.31, p = 0.048; r = −0.34, p = 0.03, respectively), and not correlated with serum IL-6 or CRP. Serum Lp-PLA_2_ activity levels were negatively correlated with IL-6 detected by IHC (r = −0.32, p = 0.04) and not correlated with plaque IL-6 expression, serum IL-6 or CRP.

**Figure 2:**
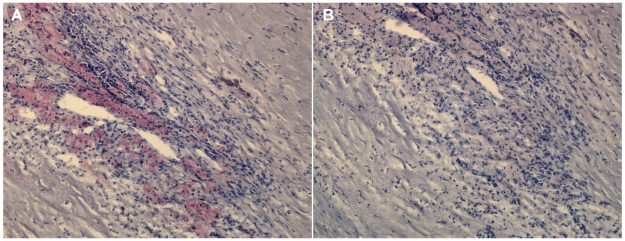
Carotid plaque section from a patient with atherosclerosis. A) Lipoprotein-associated phospholipase A2 (Lp-PLA_2_) was detected by Immunohistochemistry (IHC) using anti- Lp-PLA_2_ specific monoclonal antibody (diaDexus) and chromagen fast red; 400×. B) The five micron adjacent carotid plaque section from the same patient was stained as in A but the primary antibody was omitted as a control for specificity; 400×.

Figure 2A shows staining of Lp-PLA_2_ (red) in the perivascular necrotic area of carotid plaque. This region was rich in macrophages in addition to *C. pneumoniae* infected macrophages. Figure 2B shows the adjacent section stained with secondary antibody and omission of primary antibody against Lp-PLA_2_ as a control for specificity. There were similar results for staining with the control mouse non-immune IgG antibody (data not shown).

## Discussion

This is the first study, to our knowledge, that evaluates the correlation between Lp-PLA_2_ serum mass and activity levels with presence of Lp-PLA_2_ in carotid plaques, and the association of these indicators with plaque *C. pneumoniae* and other inflammatory mediators. Lp-PLA_2_ serum mass and activity levels correlated well with one another but not with plaque Lp-PLA_2_. However, plaque Lp-PLA_2_ was significantly correlated with plaque *C. pneumoniae* infection, macrophages and serum homocysteine levels. A high percentage of macrophages were infected, and many cells showed co-localization of Lp-PLA_2_ with *C. pneumoniae*. Thus, a possible mechanism for *C. pneumoniae* in the atherogenic process may involve infection of macrophages that induce Lp-PLA_2_ production leading to upregulation of inflammatory mediators in plaque tissue.

We found no significant correlation between patient risk factors for atherosclerosis and serum levels or plaque grade for Lp-PLA_2_ in our study. Our findings are similar to those of others [29] but in contrast to some publications that reported a correlation between clinical characteristics or risk factors and serum Lp-PLA_2_ mass or activity levels [30], [31]. Earlier publications initially found strong correlations between serum Lp-PLA_2_ levels and clinical characteristics, which decreased significantly after adjustment for measures of atherosclerosis [32], [33]. In our study, the lack of correlation between either clinical characteristics or risk factors and Lp-PLA_2_ might be explained by the small sample size. However, serum Lp-PLA_2_ mass and activity levels may not be consistently reliable risk markers for atherosclerosis.

There is prior evidence that both serum Lp-PLA_2_ mass and activity levels are influenced by infection such as hepatitis C, malaria and influenza [34], [35], [36]. For example, malaria researchers have shown a positive correlation between circulating levels of Lp-PLA_2_, parasitemia and severity of disease [35]. Studies of the interrelationship of influenza with inflammatory responses and atherosclerosis were initiated based on the observation of a strong association between acute respiratory infections, acute MI and sudden death in winter [36]. In a murine model of influenza, Lp-PLA_2_ activity in high density lipoproteins (HDL) was found to decrease two days after inoculation of influenza, reaching the lowest levels within a week, while Lp-PLA_2_ modification of LDL and lipid peroxide products increased as monocyte migration was induced [36].

In our study, only plaque Lp-PLA_2_, but not serum Lp-PLA_2_ mass or activity levels, was significantly associated with the presence of *C. pneumoniae*. Prior studies have shown that persistent *C. pneumoniae* infection, characterized by up-regulation of chlamydial heat shock protein 60 expression, induced LDL oxidation that leads to macrophage activation [37]. It is well known that oxidized LDL is also a substrate of Lp-PLA_2_ catalyzed reactions, resulting in LysoPC and oxFA [5]. LysoPC induces proinflammatory cytokines and chemokines, such as IL-1β, IL-6, TNF-α, and monocyte chemoattractant protein 1 (MCP-1) [38]. IL-1β, IL-6 and TNF-α trigger atherogenesis by sensitizing vascular smooth muscle cells [39] and inducing secretion of cellular adhesion molecules [40] and matrix metalloproteinase (MMP) by monocytes during later stages of atherosclerosis [41]. MCP-1 recruits T cells and monocytes, inhibits endothelial nitric oxide (causing endothelial dysfunction), induces monocyte-macrophage colony-stimulating factor (M-CSF) secretion by smooth muscle cells and stimulates macrophage proliferation [42], [43], [44]. In our study, we found that plaque Lp-PLA_2_ was significantly correlated with plaque macrophages, which is consistent with these studies.

Several studies have shown that *C. pneumoniae* activated macrophages induce pro-inflammatory cytokine/chemokines, such as IL-6, IL-8 and MCP-1 [18], [45], [46]. In our previous evaluation of the same tissue samples as in the present study, we found that macrophages in the carotid plaques co-localized with CD4+ lymphocytes [18], which can secrete pro-inflammatory cytokines and further fuel the atherogenic process. Both CD4+ cells and macrophages release interferon gamma (IFN-γ), which can resolve chlamydial infection or stimulate a non-replicative persistent state that can result in chronic infection that is likely resistant to antimicrobial treatment.

IL-6 is an acute phase reactant secreted by activated macrophages, Th2 cells and B cells. We previously showed that quantitatively higher levels of carotid plaque *C. pneumoniae* measured by qRT-PCR and semi-quantitative IHC was associated with higher IL-6 expression in both plaques and serum [18]. Subsequent studies have also shown that atherosclerotic progression, based on intima-media wall thickness, was associated with higher serum IL-6 levels among *C. pneumoniae* patients [47]. In *in vitro* studies, *C. pneumoniae* induces the production of IL-6 in peripheral monocytes and smooth muscle cells [45]. Neither serum nor plaque IL-6 correlated with serum Lp-PLA_2_ activity or mass levels or with plaque Lp-PLA_2_ grade in our study. However, one other study also failed to show a correlation between serum IL-6 and Lp-PLA_2_ activity [48]. This might be due to the indirect pathways induced by Lp-PLA_2_ where the temporal influence of Lp-PLA_2_ and up-regulation of serum IL-6 are missed because only a single serum sample is obtained at the time of endartectomy. Similarly, given that we found a lack of association of serum Lp-PLA_2_ mass or activity levels with plaque Lp-PLA_2_ and with plaque *C. pneumoniae*, it is possible that either the timing of sample collection yields a false negative result or that what is occurring locally in the tissue does not always reflect the circulating systemic levels of Lp-PLA_2_. Thus, some patients may not express elevated serum Lp-PLA_2_ levels in association with risk factors or disease [32], [33] or with infection.

There was a significant correlation of plaque Lp-PLA_2_ with serum homocysteine levels. Homocysteine exerts an independent effect on vascular smooth muscle cell proliferation, although the mechanism(s) is not well understood [49]. It is unclear from our data whether there is a direct interaction between homocysteine and plaque Lp-PLA_2_ that may accelerate atherosclerotic progression.

Overall, we found that macrophages, many of which were infected with *C. pneumoniae*, co-localized with Lp-PLA_2_. A high percentage of cells demonstrated co-localization of Lp-PLA_2_ and *C. pneumoniae*. These findings suggest that macrophages may be activated by *C. pneumoniae* infection, inducing Lp-PLA_2_ production and subsequent proinflammatory mediators, and, under the influence of Lp-PLA_2_ byproducts, result in macrophage proliferation that in turn release inflammatory mediators. This scenario indicates a possible indirect mechanism for *C. pneumoniae* involvement in the atherogenic process. However, additional research focused on *in vitro* cell and *in vivo* animal models will be needed to advance our understanding of the interaction of *C. pneumoniae* infection with Lp-PLA_2_ in inflammation and atherosclerotic disease.
